# Advanced Computational Intelligence for Object Detection, Feature Extraction and Recognition in Smart Sensor Environments

**DOI:** 10.3390/s21010045

**Published:** 2020-12-24

**Authors:** Marcin Woźniak

**Affiliations:** Faculty of Applied Mathematics, Silesian University of Technology, 44-100 Gliwice, Poland; marcin.wozniak@polsl.pl

## 1. Special Issue

The recent years have seen a vast development in various methodologies for object detection and feature extraction and recognition, both in theory and in practice. When processing images, videos, or other types of multimedia, one needs efficient solutions to perform fast and reliable processing. Computational intelligence is used for medical screening where the detection of disease symptoms is carried out, in prevention monitoring to detect suspicious behavior, in agriculture systems to help with growing plants and animal breeding, in transportation systems for the control of incoming and outgoing transportation, for unmanned vehicles to detect obstacles and avoid collisions, in optics and materials for the detection of surface damage, etc. In many cases, we use developed techniques which help us to recognize some special features. In the context of this innovative research on computational intelligence, contributions to the Special Issue “Advanced Computational Intelligence for Object Detection, Feature Extraction and Recognition in Smart Sensor Environments” present an excellent opportunity for the dissemination of the recent results and achievements for further innovations and development.

Among the total 88 manuscript submissions to this Special Issue, only 24 manuscripts were accepted after a rigorous reviewing process and published in final forms as a separate MDPI *Sensors* volume collection under the link https://www.mdpi.com/journal/sensors/special_issues/computational_intelligence_object_detection. This creates an acceptance rate at the level of 27.2%, which confirms the high level of presented research and the outstanding interest of researchers in contributing their innovative research articles to this venue. The published articles show innovative research results from authors from Europe, Asia, the Americas, and Africa, showing a worldwide research interest in the topic of this Special Issue and the importance of the proposed contributions. The published articles cover important fields of science and technology by showing models and applications for medical image processing, automated drone and vehicle driving systems, marine object detection and recognition, and agriculture and harvesting, with many interesting theoretical aspects of new training models and data augmentation. Additionally, the published articles bring new data sets to the scientific community—i.e., defect detection from optical fabric images and Industrial10 for industrial area image processing.

## 2. Contributions

The topic of using computer vision for autonomous driving systems, aerial vehicles, and vessel classification has been covered by many innovative ideas. In [1], a model of a system developed for the detection of flying objects for automatic drone protection systems was presented. A proposed solution is composed of a background subtraction model which cooperates with the applied model of the convolutional neural network (CNN). As a result, the system detects flying drones and provides their initial recognition to the operator. In [2], a model was proposed for ship type classification. The proposed complex neural architecture was based on a time convolutional layer model which helped to compare the extracted ship features. In [3], the authors discuss a model of vehicular traffic congestion with various approaches. As a result of this, a study presented a set of comparative results for different deep learning models. In [4], a real-time vehicle detection drone system was developed which can detect a car from a bird-view perspective. The model was based on an adapted DRFBNet300 structure. In [5], the YOLOv2 model was adapted to the task of multi-scale vehicle detection. The adopted neural network was enhanced with a proposed foreground–background imbalance estimation. Another interesting model for non-conventional vessel detection was presented in [6]. An applied system using a convolutional neural network (CNN) was trained by the Adam algorithm. The authors compared various architectures and drew conclusions for the best applications in the automatic detection system.

Among interesting propositions for potential industrial applications, we can find applications for various types of images, from object surfaces to whole-scene processing. In [7], a new approach for correlating scene images in industrial areas was discussed. In this model, a concept of a regression model of nested markers was used for viewpoints in augmented reality. As a result, the research presented a more efficient image capturing technique for industrial applications, but also a new data set called Industrial10. We kindly encourage the scientific community to adapt this data set in the research on camera pose regression methods. In [8], a model to detect surface regions of interest (ROI) in 3D was presented. As a processing mechanism, a deep convolutional neural network (CNN) modeling mechanism was adapted with the Adam training algorithm. This combination was applied in industrial processes to optimal CCD laser image scanning with very good results. In [9], an idea of composite interpolating feature pyramid (CI-FPN) was applied in a model of fabric defect detection. The result was processed by a cascaded guided-region proposal network (CG-RPN) to classify the detected regions. In addition to the model, this research article also introduced a new data set for defect detection from optical fabric images. In [10], a model of a convolutional neural network (CNN) for industrial application in tool wear identification was presented, where parts of the face milling process can be evaluated for potential damage. An application in farming and plant growing was proposed in [11]. A proposed model of a weakly dense connected convolution network (WeaklyDenseNet-16) was used to detect plant disease from images. In [12], a system model for robotic inspection tasks was proposed. The proposed system enabled drones to detect novelty in inspected areas from a distant viewpoint.

This Special Issue also received interesting research presentations concerning human pose detection and recognition. In [13], an innovative video frame analysis model for surveillance and security applications was presented. The model uses a support vector machine (SVM) or a convolutional neural network (CNN) as an extractor and detector of key features from CCTV and operation units. As a result, a faster detection of potential situations for legal actions was achieved. In [14], a model of active player detection for a sport vision system was presented. The solution was based on the idea of a bounding box area, which was associated with motion centroids of the human body pose. As a result, a model of active support for sport transmission to annotate players during the game was developed. In [15], a hand gesture recognition model was proposed. Such a development can be very useful for a man–machine interaction system, where the computer should read human intention, i.e., from the hand gesture presented to the camera. The proposed model was based on EMGNet architecture processing images collected by using electronic marker devices such as the Myo armband.

Another important category is new models of image processing and feature extraction and detection by the developed models of computational intelligence. In [16], a new approach to remote sensing image processing was presented, where the image should be cleared from radio-frequency interference (RFI) artefacts. The model used a proposed pixel value conversion from RGB to greyscale as a means to detect such artifacts and remove them from the adapted neural network. In [17], a semantic segmentation approach to object extraction from images was examined. The model proposed adapted the WASPnet architecture working on the Waterfall Atrous Spatial Pooling (WASP) module. Experiments showed a high efficiency for various types of images. In [18], a comparative review for models of traffic sign detection systems based on various computational intelligence techniques was presented.

The Special Issue received several interesting articles in the domain of medical image processing, where new ideas proposed models of detection and recognition of tissue features. In [19], an applied model of a SegNet convolutional neural network encoder-decoder construction used for more efficient medial image processing was presented. As a result, a processing model for tumor segmentation in CT liver scans in a DICOM format was proposed. In [20], a model for human embryo image generator based on generative adversarial networks (GAN) trained using the Adam algorithm was proposed. The resulting model enables one to manipulate the size, position, and number of artificially generated embryo cells in the composed image. In [21], acute brain hemorrhages on computed tomography scans were detected with the use of an adapted 3-dimensional convolutional neural network. The main goal of such a system is to efficiently reduce the time between diagnosis and treatment.

The Special Issue also received some interesting propositions for various pattern analysis. In [22], a simulation result for vibration signals of high-speed trains for non-stationary object modeling was presented. The research presents the use of intelligent modeling for signal noise reduction. The model proposed in [23] discussed an idea for information retrieval from large-scale text data by using BERT (CLS) representation. To improve this, an efficiency method was based on reasoning paths from a composed cognitive graph structure. In [24], a multi-view approach was discussed for visual question answering (VQA) systems, which are encountered in complex artificial intelligence systems, as operating both in text conversation and image processing and recognition. The proposed approach gave us a chance to boost such systems due to processing several images from one scene, and this therefore enabled the system to consider more aspects on the way to a final decision.

In summary, we should congratulate the authors of the articles accepted in this Special Issue for their outstanding research results and wish them great success in the continuation of their research and projects for future development. The topic of the Special Issue was clearly well accepted in the worldwide scientific community, which gives a sign for the future research and direction of trends for technology and science in the field of computational intelligence for object detection, feature extraction, and recognition in smart sensor environments.
